# Administration of probiotics influences F4 (K88)-positive enterotoxigenic *Escherichia coli *attachment and intestinal cytokine expression in weaned pigs

**DOI:** 10.1186/1297-9716-42-69

**Published:** 2011-05-23

**Authors:** Jean-François Daudelin, Martin Lessard, Frédéric Beaudoin, Éric Nadeau, Nathalie Bissonnette, Yvan Boutin, Jean-Philippe Brousseau, Karoline Lauzon, John Morris Fairbrother

**Affiliations:** 1 Dairy and Swine Research and Development Centre, Agriculture and Agri-Food Canada, 2000 College Street, Sherbrooke, Quebec, J1M 1Z3, Canada; 2 Université de Montréal, Faculté de médecine vétérinaire, Escherichia coli Laboratory, 3200 Sicotte Street, Saint-Hyacinthe, Quebec, J2S 7C6, Canada; 3 Transbiotech, 201 Monseigneur-Bourget Street, Lévis, Quebec, G6V 9V6, Canada

## Abstract

This study evaluated the effect of the probiotics *Pediococcus acidilactici *and *Saccharomyces cerevisiae boulardii *on the intestinal colonization of O149 enterotoxigenic *Escherichia coli *harbouring the F4 (K88) fimbriae (ETEC F4) and on the expression of ileal cytokines in weaned pigs. At birth, different litters of pigs were randomly assigned to one of the following treatments: 1) control without antibiotics or probiotics (CTRL); 2) reference group in which chlortetracycline and tiamulin were added to weanling feed (ATB); 3) *P. acidilactici*; 4) *S. cerevisiae boulardii*; or 5) *P. acidilactici *+ *S. cerevisiae boulardii*. Probiotics were administered daily (1 × 10^9 ^CFU per pig) during the lactation period and after weaning (day 21). At 28 days of age, all pigs were orally challenged with an ETEC F4 strain, and a necropsy was performed 24 h later. Intestinal segments were collected to evaluate bacterial colonization in the small intestine and ileal cytokine expressions. Attachment of ETEC F4 to the intestinal mucosa was significantly reduced in pigs treated with *P. acidilactici *or *S. cerevisiae boulardii *in comparison with the ATB group (*P *= 0.01 and *P *= 0.03, respectively). In addition, proinflammatory cytokines, such as IL-6, were upregulated in ETEC F4 challenged pigs treated with *P. acidilactici *alone or in combination with *S. cerevisiae boulardii *compared with the CTRL group. In conclusion, the administration of *P. acidilactici *or *S. cerevisiae boulardii *was effective in reducing ETEC F4 attachment to the ileal mucosa, whereas the presence of *P. acidilactici *was required to modulate the expression of intestinal inflammatory cytokines in pigs challenged with ETEC F4.

## Introduction

Antimicrobials are commonly used as growth promoters and to prevent or treat gastrointestinal infections in weaned pigs. In North America, postweaning diarrhea is a major health problem in swine that results in significant financial losses in pig production. Infection with enterotoxigenic *Escherichia coli *harbouring the F4 (K88) fimbriae (ETEC F4) is one of the most important causes of postweaning diarrhea in pigs [1]. This pathotype is characterized by the expression of an F4 fimbrial adhesin which induces bacterial attachment to specific F4 receptors located in the brush border of the swine intestine and secretion of enterotoxins that cause diarrhea [1]. The emergence of antimicrobial resistance in the pig intestinal microflora and the risk of resistance gene transfer to pathogenic bacteria in humans have increased the pressure on pig producers to seek alternative strategies. Among the strategies that have been proposed, the use of probiotics has become quite attractive because of their potential to stimulate the intestinal immune system and to increase the production of antimicrobial peptides and cytokines in the intestinal tract [2,3].

Some pig producers currently use probiotics to reduce antimicrobial use. For instance, the administration of lactic acid bacteria such as *Pediococcus acidilactici *or yeasts such as *Saccharomyces cerevisiae boulardii *improves intestinal defences against microbial infection and increases performance in different species of monogastric animals; *P. acidilactici *was shown to improve weight gain and reduce oocyst shedding in chickens challenged with coccidia [4] and *S. cerevisiae boulardii *was reported to improve postweaning daily weight gain in pigs and to increase the number of mucosal macrophages [5]. Furthermore, Di Giancamillo et al. [6] demonstrated that the administration of *P. acidilactici *positively influences weight gain and results in increased villus height and crypt depth in weaned pigs. In a previous study, the present authors also observed, following an ETEC F4 challenge, a decrease in bacterial translocation to the mesenteric lymph nodes (MLN) in weaned pigs treated with *P. acidilactici*, *S. cerevisiae boulardii *or both probiotics [7], as well as a reduction in bacterial diversity in the intestine of pigs treated with *P. acidilactici *after weaning [8,9].

The aim of the present study was to determine the effect of *P. acidilactici*, *S. cerevisiae boulardii *or a combination of both probiotics on the consistency of the intestinal contents, intestinal colonization and attachment of ETEC F4 bacteria, and expression of intestinal cytokines after an ETEC F4 challenge of weaned pigs and to evaluate the potential for use of these probiotics as an alternative to antibiotics added to the weanling diet.

## Materials and methods

### Animals and treatments

The animal use protocol was reviewed and approved by the Dairy and Swine Research and Development animal care committee and followed the principle established by the Canadian council on animal care [10]. A total of 40 Yorkshire-Landrace gilts obtained from La Coop fédérée (Montreal, QC, Canada) and housed at Agriculture and Agri-Food Canada's Dairy and Swine Research and Development Centre (Sherbrooke, QC, Canada) were used to carry out the experiment. Two batches of 20 gilts were constituted and each batch of gilts was used in two successive parities over a two-year period. Among a total of expected 80 litters, 40 were used as described below. Regu-Mate (Intervet Canada Ltd., Whitby, ON, Canada) was used to synchronize oestrus, and sows were inseminated twice with the same tested semen provided by the Centre d'insémination porcine du Québec inc. (St-Lambert-de-Lauzon, QC, Canada).

Twenty-eight days before parturition (day -28), pregnant sows were allocated to one of the five treatment groups using a complete randomized block design. Three groups of sows and their litters were assigned to one of the following treatments: *P. acidilactici *(strain MA18/5M, Lallemand Animal Nutrition, Blagnac, France), *S. cerevisiae boulardii *(strain SB-CNCM I-1079, Lallemand Animal Nutrition), or *P. acidilactici *in combination with *S. cerevisiae boulardii*. The other two groups were used as reference and control groups respectively; piglets of the reference group received at weaning a diet medicated with chlortetracycline and tiamulin antibiotics (ATB) and those of the untreated control group (CTRL) were fed weanling basal diet without ATB or probiotics. For both groups, sows and their litters remained untreated throughout the gestation and lactation periods. The ATB group was included as a reference group because antibiotics are commonly added to the weanling feed of pigs in North America. The sows assigned to the probiotic treatments received 2.5 × 10^9 ^CFU from day -28 to day -14, 3.5 × 10^9 ^CFU from day -14 to day 0 (parturition), and 6 × 10^9 ^CFU from day 0 to day 21. In the *P. acidilactici *+ *S. cerevisiae boulardii *group, both probiotics were simultaneously given at the indicated concentrations above. The probiotic doses were mixed with 500 g of feed and given to the sows before the morning meal. The daily feed ration given to the sows was 2.5 kg from day -28 to day -14, 3.5 kg from day -14 to day 0, and ad libitum during the lactation period (day 0 to day 21). The groups of sows were housed in different pens located in different sections of the gestation room to prevent cross-contamination between the treatments. One week before farrowing, the sows were transferred to rooms allocated to different treatments in the maternity section. From each batch of 20 gilts, 10 gilts and their piglets (two litters per treatment) were randomly chosen. Within the first 24 h after parturition, litter size was adjusted to 12 piglets and, if necessary, adoptions were carried out from litters assigned to the same treatment.

Twenty-four hours after birth, the pigs started to receive orally the same probiotic treatment as their mother by means of disposable pipettes. In the probiotic groups, the daily dose of each probiotic was 1 × 10^9 ^CFU diluted in 2 mL peptone water. The pigs in both the control and reference groups (CTRL and ATB, respectively) received 2 mL peptone water alone. The probiotics or peptone water were given daily during lactation and after weaning at 21 days of age (day 21) until the last day of the experiment (the challenge period). At weaning, all pigs were transferred to their respective pens to prevent cross-contamination. Pigs having received probiotics during the lactation were also fed a basal diet enriched with the same probiotic at 2 × 10^9 ^CFU/kg. The pigs in the ATB group received the same diet supplemented with chlortetracycline (110 ppm active ingredient/kg) and tiamulin (31.2 ppm active ingredient/kg). The basal diet was provided by La Coop fédérée (Table 1). Feed and water were available ad libitum to the weaned pigs.

**Table 1:** Nutrient composition of the weaning diet (day 21 to 31).

Nutrient	Concentration
Protein (Corn, wheat and soybean meal), %	21.5
Fat, %	7.52
Fiber, %	2.34
Calcium, %	1.00
Phosphorus, %	0.76
Sodium, %	0.2
Copper, mg/kg	128.17
Zinc, mg/kg	138.41
Vitamin A, IU/kg	11,500
Vitamin D, IU/kg	1,140
Vitamin E, IU/kg	56

### DNA marker-based test for detection of F4 receptor genes

Jugular blood samples were collected from the pigs at five days of age (day 5). The test for the detection of the F4 receptor genes was performed as described by Jensen et al. with minor modifications [11]. Briefly, DNA was extracted from 100 μL blood using the QIAamp DNA Mini Kit (Qiagen Inc., Mississauga, ON, Canada). The polymerase chain reaction-restriction fragment length polymorphism (PCR-RFLP) assay was performed on 20 ng genomic DNA from each pig in a total volume of 20 μL using 1X Standard Taq Buffer (New England Biolabs Inc., Pickering, ON, Canada), 200 μM of each deoxyribonucleotide triphosphate (dNTP), 5.0 μM of each primer (5'-GTGCCTTGGGTGAGAGGTTA/5'-CACTCTGCCGTTCTCTTTCC), and 1 U Taq DNA polymerase (New England Biolabs). The thermocycling conditions were 5 min of initial denaturation at 95°C, followed by 95°C for 30 s in the additional cycles. Extension was carried out at 72°C for 1 min. Touchdown was performed by lowering the annealing temperature by 1°C after each cycle in the first 10 cycles, starting at 59°C. The last 30 cycles were performed at an annealing temperature of 49°C. This test relies on an *Xba*I-polymorphism in intron 7 of the porcine *Sus scrofa *mucin 4 (*MUC4*) gene (Genbank accession no. GU983681). Ten μL of the 367 bp PCR product were used for digestion with *Xba*I (Invitrogen Canada Inc., Burlington, ON, Canada), as recommended by the supplier. The resistant allele is not digested by *Xba*I, whereas the susceptible allele is digested into 151- and 216-bp fragments. The PCR-RLFP assay permitted discrimination between F4 receptor positive (F4R^+^) and negative (F4R^-^) pigs. Blood from gilts was also examined by the PCR-RFLP and, when possible, F4R^+ ^gilts were selected. On the other hand, boars from which semen was collected were not examined by the PCR RFLP and, therefore, susceptible heterozygous and homozygous F4R^+ ^pigs were used for the challenge experiment (eight pigs per treatment). As all piglets of one litter of the *S. cerevisiae boulardii *group were F4R^-^, only seven pigs instead of eight were challenged for this group.

### *E. coli *strain

The ETEC F4 strain ECL8559 (0149:LT:STa:STb:East1:paa:hemβ:F4) used in the challenge study was isolated at the *E. coli *Laboratory at the Faculté de médecine vétérinaire (Saint-Hyacinthe, QC, Canada) of the Université de Montréal from the feces of a sick 42-day-old pig. A nalidixic acid-resistant (Nal^r^) variant of this strain was obtained by serial passage in Luria-Bertani (LB) broth containing concentrations of nalidixic acid from 0 to 60 μg/mL at 37°C for 24 h. This Nal^r ^variant was used in the challenge studies. Moreover, this ETEC F4 strain showed acquired resistance to different antibiotics, including chlortetracycline and tiamulin.

### ETEC F4 challenge

At 25 days of age, one F4R^+ ^pig from each litter was transferred to a level 2 biosecurity containment facility (Canadian Food Inspection Agency, Saint-Hyacinthe Laboratory, QC, Canada). The pigs were housed in groups (one pen per treatment group). Three days after transfer (day 28), the 10 pigs were orally challenged with 1 × 10^9 ^CFU ETEC F4 strain ECL8559 in 5 mL Trypticase soy broth (Difco Laboratories, Inc., Detroit, MI, USA) following the administration of 10 mL CaCO_3 _1.2% to neutralize gastric acid. Both inocula were administered through an intra-oesophageal tube. The pigs were evaluated for general appearance, attitude, dehydration, food and water intake, and presence of diarrhea, in accordance with the guidelines of the Canadian Council on Animal Care, and were euthanized 24 h post-challenge (hpc), on day 29. Necropsies were performed, and intestinal contents and various tissues were collected always at the same location for all the pigs for further analysis. The non-challenged pigs, (one randomly chosen pig per litter) receiving the same treatments and kept at the Dairy and Swine Research and Development Centre, were euthanized on day 31.

### Microbiological analysis of intestinal contents, ileal mucosa, and MLN

Following euthanasia, intestinal contents from the ileum and proximal colon were sampled for enumeration of the probiotics and of the ETEC F4 challenge strain. Plates containing LAMVAB agar [12] were used for the detection of the bacterium *P. acidilactici*. The plates were incubated aerobically for 48 h at 37°C. Potato dextrose agar (BD Biosciences, Mississauga, ON, Canada) was used for the detection of the yeast *S. cerevisiae boulardii*. To inhibit bacterial growth, the medium was acidified by adding sterile tartaric acid (10% solution) until a pH of 3.5 was reached. The yeast plates were incubated at 30°C for 48 h. To enumerate the ETEC F4 strain in the intestinal contents, samples were serially diluted and plated on MacConkey agar No. 2 (Oxoid Company, Nepean, ON, Canada) supplemented with nalidixic acid at 50 μg/mL (Sigma-Aldrich Canada Ltd., Oakville, ON, Canada). The plates were incubated aerobically for 24 h at 37°C.

For the detection of viable ETEC F4 on the ileal mucosa and in ileocecal MLN, 300-400 mg of tissue, lightly washed in phosphate-buffered saline (PBS), was homogenized for 5 s using a Polytron homogenizer (Kinematica Inc., Bohemia, NY, USA) in 3-4 mL sterile PBS.

### Intestinal content consistency scores

Ileal, cecal and colon content consistency scores, representing fluid accumulation in the intestine, were assigned by the same person and assessments were performed in a blinded fashion. Consistency scores were ranked using the following scale: 0, normal, solid contents; 1, soft intestinal contents, and looser contents than normal; 2, semi-liquid intestinal contents; and 3, liquid contents.

### ETEC F4 attachment to ileal mucosa

**Figure 1:**
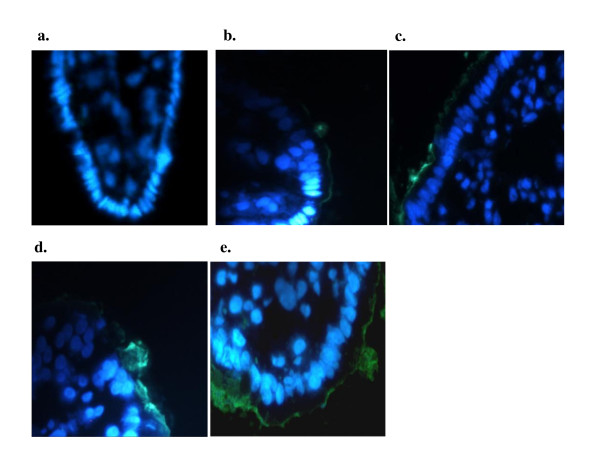
**Detection of adherent ETEC F4 bacteria on ileal sections of challenged F4-receptor positive pigs using the immunofluorescent assay**. Green staining (fluorescein isothionate) represented ETEC F4 bacteria and nuclei of epithelial cells were stained with 5 μg/mL of DAPI (blue). Representative ileal sections with an attachment rating of 0 (A), 1 (B), 2 (C), 3 (D) and 4 (E).

The attachment of ETEC F4 to the ileal mucosa was determined using an indirect immunofluorescence assay (IFA). At necropsy, two ileal segments (between 15-20 cm from the ileocecal valve) from each pig were embedded in Tissue-Tek O.C.T. compound (Sakura Finetek USA, Inc., Torrance, CA, USA) and immediately frozen in liquid nitrogen. Frozen ileal segments were subsequently sectioned at 5 μm using a Leica CM3050 cryostat (Leica Microsystems Inc., Richmond Hill, ON, Canada), fixed in 100% methanol for 5 min, and treated with a blocking solution containing 3% bovine serum albumin (Sigma-Aldrich Canada) for 45 min at 37°C. The sections were then incubated with a 1:100 dilution of rabbit anti-F4 fimbriae antiserum (provided by the *E. coli *Laboratory) for 45 min at 37°C. After several washes in PBS, the sections were rinsed with PBS and incubated with goat anti-rabbit fluorescein isothiocyanate-conjugated (FITC) secondary antibody (Jackson ImmunoResearch Laboratories, Inc., West Grove, PA, USA) for 45 min at 37°C. The DNA of epithelial cells was counterstained with 4', 6-diamidino-2-phenylindole dilactate (DAPI) at 5 μg/mL (Invitrogen Canada). The sections were mounted and examined in a blinded fashion with a Leica DMR microscope (Leica Microsystems) equipped with epifluorescence and UV excitation modules. Attachment was rated from 0, indicating no observed attachment of ETEC F4 to the ileal mucosa, to 4, indicating that several layers of ETEC F4 were attached to the entire villous length (Figure 1). One ileal section per pig was analyzed and five randomly selected fields were visualized for each ileal section.

### RNA extraction and cDNA synthesis

At necropsy, the ileal slices were immediately frozen in liquid nitrogen and stored at -80°C until RNA extraction and cDNA synthesis were performed. Briefly, the ileal slices were homogenized in TRIzol reagent (Invitrogen Canada) using a Polytron homogenizer (Kinematica). Total RNA was extracted following the manufacturer's recommendations. The RNA was resuspended in 50 μL ultrapure water (Invitrogen Canada) containing SUPERase•In at 1 U/μL (Applied Biosystems Inc., Foster City, CA, USA). Total RNA was quantified using a NanoDrop spectrometer (NanoDrop Technologies, Inc., Wilmington, DE, USA) at a wavelength of 260 nm. The RNA integrity was confirmed by examination of the presence of the 18S and 28S ribosomal bands on agarose gels stained with ethidium bromide before proceeding to gene expression analysis. Aliquots of RNA were treated with DNase I (Invitrogen Canada) and precipitated in 100 μL containing 50 μL ammonium acetate (7.5 M) (Sigma-Aldrich Canada), 3 μL linear acrylamide (5 mg/mL) (Applied Biosystems), and 300 μL anhydrous ethyl alcohol (200 HP) (Commercial Alcohols Inc., Brampton, ON, Canada). Purity was assessed by determining the ratio of absorbance at 260 and 280 nm (A_260_/A_280_). All samples had a ratio between 1.7 and 2.0. A 1 μg aliquot of total RNA was reverse-transcribed with Superscript II reverse transcriptase (Invitrogen Canada) using oligo(dT)_12-18 _primer (Invitrogen Canada) in a final volume of 20 μL, according to the supplier's instructions. The cDNA samples were diluted 1:20 in nuclease-free water and aliquots were stored at -20°C prior to real-time PCR analysis. All the cDNA samples in this experiment were analyzed for the expression of three different reference genes: *β-actin *(*ACTB*), glyceraldehyde-3-phosphate dehydrogenase (*GAPDH*) and cyclophilin A (*PPIA*). These three reference genes are commonly used in in vivo experiments similar to that of our study [13-15]. Statistical analyses were performed and results indicated that *ACTB *was the most stable reference gene for use in our experiment, as its expression was not altered by the treatments administered to pigs in contrast to the two other tested reference genes (data not shown).

### Quantification of cytokine gene expression by real-time PCR

Real-time PCR was performed with a 7500 Fast Real-Time PCR System (Applied Biosystems) to evaluate the expression of the cytokines listed in Table 2. The PCR mixture was composed of the following: 5 μL *Power *SYBR Green Master Mix (Applied Biosystems), 0.1 μL AmpErase uracil-N-glycosylase (Applied Biosystems), each set of gene specific primers (Applied Biosystems) at the indicated concentrations (Table 2), 2 μL of diluted 1:20 cDNA and completed to a final volume of 10 μL with molecular grade water (Invitrogen Canada). The primers were designed using the IDT SciTools PrimerQuest software [16] and selected using the following criteria, when possible: (1) both forward and reverse primers encompass two consecutive exons; and (2) no more than two guanines or cytosines within the last five nucleotides in the 3' termini. The PCR cycling conditions used were in accordance with the manufacturer's protocol. The level of expression was determined using a standard curve established by serial dilution of a DNA construct for the respective cloned gene. These gene constructs were created by cloning gene-specific cDNA PCR products using the TA Cloning Kit (Invitrogen Canada) according to the manufacturer's instructions. Normalization of the target gene expression level was performed by dividing the target copy number by the *ACTB *copy number. In order to confirm the specificity of the measured amplicons (i.e. the presence of one amplicon), the melting curve was systematically analyzed for all samples. Each run included a no-template control to detect DNA contamination of the reagents and each reaction was performed in triplicate.

**Table 2:** List of genes and sequences of the primers used for real-time PCR^1^.

mRNA target	**Primers (5' → 3')**^**2**^	Product size (bp)	**Final concentration (nM)**^**3**^
IL-4	F:GGTCTGCTTACTGGCATGTACC	117	150
	R:CTCCATGCACGAGTTCTTTCTC		150
IL-6	F:GGAAATGTCGAGGCTGTGCAGATT	87	300
	R:GGTGGTGGCTTTGTCTGGATTCTT		300
IL-8	F:AGAACTGAGAAGCAACAACAACAG	132	300
	R:CACAGGAATGAGGCATAGATGTAG		300
IL-10	F:GATATCAAGGAGCACGTGAACTC	137	300
	R:GAGCTTGCTAAAGGCACTCTTC		300
IL-12p35	F:TGCAGGCTCTGAATTTCAAC	111	150
	R:CACGAATTCTGAAGGCATGA		150
IL-12p40	F:CTTCATCAGGGACATCATCAAAC	196	300
	R:GGTCCGTGAAGAGTTTATCTTTCT		150
IFN-γ	F:AGGTTCCTAAATGGTAGCTCTGGG	101	300
	R:AGTTCACTGATGGCTTTGCGCT		300
TNF-α	F:CACTGACCACCACCAAGAATTGGA	94	300
	R:CATTCCAGATGTCCCAGGTTGCAT		300
pBD-2	F:CCGACCACTACATATGTGCCAAGA	93	300
	R:TGCCACTGTAACAGGTCCCTTCAA		300
COX-2	F: AAGCGAGGACCAGCTTTCACCAAA	93	300
	R:GCGCAGTTTATGCTGTCTCTCCAA		300
*ACTB*	F:CTCTTCCAGCCCTCCTTCCT	104	300
	R:GCGTAGAGGTCCTTCCTGATGT		300

^1^IL = interleukin; IFN = interferon; TNF = tumor necrosis factor; pBD-2 = porcine β-defensin-2; COX-2 = cyclooxygenase-2; *ACTB *= *β-actin*.

^2^F and R indicate forward and reverse primers, respectively.

^3^Final concentration of forward (F) and reverse (R) primers.

### Statistical analysis

Data were analyzed as a randomized complete block design with the litter as the experimental unit. The MIXED procedure of SAS (SAS Institute, Inc., Cary, NC, USA) was used to perform statistical analyses on the different variables, and the model included the treatment effect (five groups). Treatment comparisons were done by testing the probiotic treatments (three groups) against the control and reference groups (CTRL and ATB) using a Dunnett's test for multiple testing. Comparisons were also done between challenged (day 29) and non-challenged (day 31) pigs. Consistency score data were analyzed using the Cochran-Mantel-Haenszel test, a non-parametric method for testing equality of population medians among groups.

## Results

### Reduced attachment of ETEC F4 to the ileal mucosa of weaned pigs with administration of *P. acidilactici *or *S. cerevisiae boulardii*

**Figure 2:**
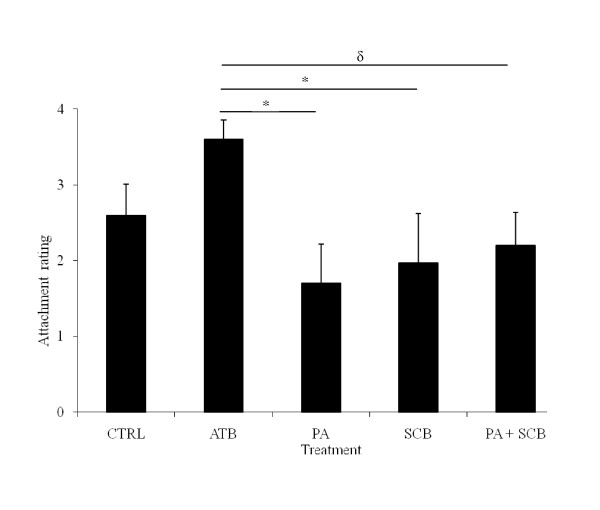
**Attachment of ETEC F4 bacteria to the ileal mucosa following challenge in F4-receptor positive pigs receiving no antibiotic or probiotic (CTRL), antibiotics (ATB), *P. acidilactici *(PA), *S. cerevisiae boulardii *(SCB) or both, PA + SCB**. Results (Bars ± SEM) are presented as the mean attachment rate evaluated using the immunofluorescent assay for six pigs per treatment, except for CTRL and ATB, where five pigs were used. * = In PA and SCB treated pigs, ETEC F4 attachment was reduced compared to ATB at *P *< 0.05. δ = In pigs treated with PA + SCB, ETEC F4 attachment tended to be reduced compared to ATB (*P *= 0.06).

The consistency scores of the ileal, cecal and colon contents 24 hpc with an ETEC F4 strain were not affected by probiotic or antibiotic treatments, most animals showing semi-liquid or liquid ileal content consistency (Table 3). However, the attachment of ETEC F4 to the ileal mucosa, as demonstrated by IFA (Figure 1), was significantly lower for the pigs receiving *P. acidilactici *or *S. cerevisiae boulardii *(*P *= 0.01 and *P *= 0.03, respectively) than for those of the ATB group but not in comparison with the CTRL group (Figure 2). Moreover, ETEC F4 attachment to the ileal mucosa tended to be reduced for pigs treated with *P. acidilactici *+ *S. cerevisiae boulardii *in comparison with those receiving ATB (*P *= 0.06). No difference between the CTRL and ATB groups was observed.

**Table 3:** Content consistency in the ileum and colon and presence of ETEC F4 in different tissues of F4-receptor-positive pigs treated with probiotics or antibiotics 24 h post-challenge with ETEC F4^1^.

	Treatments					
		
	CTRL	ATB	PA	SCB	PA + SCB	**SEM**^**2**^
Ileum content^3^	7.49	7.72	7.07	7.37	7.98	0.44
Ileum mucosa^3^	6.24	6.98	6.17	6.53	6.52	0.55
Colon content^3^	7.41	7.72	7.52	7.66	8.31	0.50
MLN^3^	3.88	4.16	3.58	3.54	4.59	0.27
Ileal consistency score^4^	3.0	2.5	2.5	2.0	2.0	--
Colon consistency score^4^	1.0	1.0	1.0	1.0	1.0	--

^1^Definition of acronyms: CTRL = No probiotic and no antibiotic; ATB = tiamulin and chlortetracycline; PA = *P. acidilactici*; SCB = *S. cerevisiae boulardii*; PA + SCB = a mixture of the two probiotics.

^2^Pooled standard error of the mean (SEM); *n *= 8/treatment, except for SCB, *n *= 7.

^3^Results are expressed as Log_10_CFU of ETEC F4/g of tissue.

^4^Results are expressed as the median. Scale used was: 0 = normal, solid contents; 1 = soft intestinal contents; 2 = semi-liquid intestinal contents; and 3 = liquid conten

Despite the differences observed by IFA (Figure 2), the treatments had no effect on intestinal colonization by ETEC F4 or on ETEC F4 absolute count in the ileum, colon and MLN (Table 3). Finally, in our experimental conditions, ETEC F4 challenge did not influence *P. acidilactici *or *S. cerevisiae boulardii *absolute count in the ileum or colon of pigs treated with either probiotic or both in comparison to non-challenged pigs of the probiotic groups (data not shown).

### Modulation of cytokine gene expression by probiotics in the ileum of ETEC F4-challenged weaned pigs

The gene expression levels of proinflammatory cytokines in the ileum of the non-challenged and ETEC F4-challenged pigs are summarized in Table 4. After challenge, the expression of interleukin (IL)-6 in the ileal tissue was significantly higher in the *P. acidilactici *+ *S. cerevisiae boulardii *pigs than in the CTRL group (*P *= 0.04) and tended to be increased in comparison with the ATB group (*P *= 0.07). Similarly, a tendency toward an upregulation of IL-6 was also observed in the *P. acidilactici *group in comparison with the CTRL (*P *= 0.06).

**Table 4:** Cytokines mRNA expression levels normalized to *ACTB *in non-challenged and 24 hpc ETEC F4 pigs treated with probiotics or antibiotics^1^.

Gene	Mean cytokine mRNA expression level in the ileum					*P*-value		
	
	CTRL	ATB	PA	SCB	PA + SCB	T	Ch	T×Ch
***IL-6 *(10**^**-4**^**)**								
24 hpc	0.9^ac ^ [0.3-3.1]	1.1^ac ^ [0.3-3.6]	3.5^d ^ [1.1-11.3]	0.6 [0.2-1.9]	3.9^b ^ [1.2-12.7]	0.33	0.47	0.29
Not Ch	0.8 [0.2-3.2]	1.7 [0.4-7.4]	0.9 [0.2-3.8]	1.2 [0.3-5.0]	1.2 [0.3-5.0]			
***TNF-α-*(10**^**-3**^**)**								
24 hpc	1.28^c ^ [0.38-4.32]	2.94 [0.94-9.19]	3.87 [1.24-12.09]	1.65 [0.49-5.56]	5.28^d ^ [1.69-16.48]	0.24	0.59	0.85
Not Ch	1.59 [0.46-5.52]	4.40 [1.10-17.52]	4.53 [1.31-15.73]	3.04 [0.88-10.54]	3.03 [0.87-10.51]			
***pBD-2***								
24 hpc	0.025^c ^ [0.013-0.05]	0.045 [0.024-0.085]	0.053^d ^ [0.028-0.1]	0.05 [0.025-0.1]	0.041 [0.021-0.077]	0.33	0.83	0.88
Not Ch	0.027^**c **^ [0.01-0.068]	0.040 [0.015-0.11]	0.034 [0.013-0.088]	0.065^d ^ [0.026-0.17]	0.043 [0.017-0.11]			
***IL-12p35***								
24 hpc	0.018^c ^ [0.007-0.046]	0.029 [0.012-0.069]	0.059^d ^ [0.025-0.142]	0.017 [0.006-0.042]	0.027 [0.011-0.065]	0.31	0.59	0.65
Not Ch	0.011 [0.003-0.038]	0.033 [0.008-0.13]	0.022 [0.006-0.074]	0.025 [0.007-0.085]	0.025 [0.007-0.091]			
***IL-8***								
24 hpc	11.59 [1.29-104,14]	16.86 [2.12-133.91]	6.46 [0.81-51.3]	6.41 [0.71-57.63]	10.08 [1.27-80.04]	0.48	0.003	0.68
Not Ch	0.59^a ^ [0.08-4.47]	3.79^b ^ [0.44-32.9]	1.62 [0.21-12.36]	3.12^b ^ [0.41-23.73]	3.55^b ^ [0.44-28.63]			
***IFN-γ-*(10**^**-4**^**)**								
24 hpc	0.6 [0.1-2.7]	0.9 [0.2-3.7]	0.4 [0.1-1.7]	0.9 [0.2-3.7]	1.6 [0.4-6.6]	0.26	0.07	0.88
Not Ch	2.9 [0.6-13.5]	1.8 [0.3-9.2]	0.7 [0.2-3.5]	1.2 [0.3-5.7]	3.0 [0.6-14.1]			
***IL-10***								
24 hpc	0.2 [0.05-0.77]	0.29 [0.08-1]	0.22 [0.06-0.77]	0.09 [0.02-0.32]	0.45 [0.13-1.57]	0.39	0.85	0.47
Not Ch	0.15 [0.05-0.45]	0.38 [0.12-1.25]	0.13 [0.04-0.37]	0.25 [0.08-0.72]	0.24 [0.08-0.73]			

^1^Definition of acronyms: CTRL = No probiotic and no antibiotic; ATB = tiamulin and chlortetracycline; PA = *P. acidilactici*; SCB = *S. cerevisiae boulardii*; PA + SCB = a mixture of the two probiotics; IL = interleukin; IFN = interferon; TNF = tumor necrosis factor; pBD-2 = porcine β-defensin-2; COX-2 = cyclooxygenase-2; T = Treatment effect; Ch = ETEC challenge effect.

^2^Data are presented as the mean of target copy number/*ACTB *copy number with the range of values for a 95% confidence interval within brackets.

^a, b^Means within rows are significantly different (*P *≤ 0.05).

^c, d^Means within rows tend to be different (*P *≤ 0.10).

Challenge group: *n *= 8/treatment, except for *S. cerevisiae boulardii *and CTRL group, *n *= 7.

Not challenged group: *n *= 8/treatment, except for ATB group, *n *= 7.

In addition, trends toward increased cytokine expression in the ileum were observed for tumour necrosis factor-α (TNF-α) (*P. acidilactici *+ *S. cerevisiae boulardii *vs. CTRL; *P *= 0.08), IL-12p35 (*P. acidilactici *vs. CTRL; *P *= 0.07), and for porcine β-defensin 2 (pBD-2) (*P. acidilactici *vs. CTRL; *P *= 0.09). In contrast, no differences between groups were observed in the ileal expression of the interferon-γ (IFN-γ), IL-10 and IL-8 genes after ETEC F4 challenge. Moreover, the IL-4, IL-12p40 and cyclooxygenase-2 (COX-2) genes were weakly expressed or could not be detected 24 hpc in the ileum of the pigs (data not shown).

### Modulation of IL-8 and IFN-γ gene expression in the ileum of weaned pigs after ETEC F4 challenge

Expression of IL-8 in the ileum was significantly greater in the pigs challenged with ETEC F4 than in the non-challenged animals (*P *= 0.003) (Table 4). However, in non-challenged pigs treated with *S. cerevisiae boulardii *or *P. acidilactici *+ *S. cerevisiae boulardii*, IL-8 gene expression was significantly increased compared to the pigs in the CTRL group (*P *= 0.04). Finally, the ileal expression of pBD-2 tended to be greater in non-challenged pigs treated with *S. cerevisiae boulardii *than in the CTRL pigs (*P *= 0.09).

In contrast, the expression of IFN-γ in the ileum tended to be lower in the challenged pigs than in the non-challenged pigs (*P *= 0.07). No other effects of ETEC F4 infection were observed on the expression of the other tested cytokines.

## Discussion

The aim of this study was to evaluate the impact in weaned pigs of administering the probiotics *P. acidilactici*, *S. cerevisiae boulardii *or both on the early events following ETEC F4 challenge, including intestinal colonization and attachment of ETEC F4 and the expression of intestinal cytokines involved in the activation and regulation of the innate and acquired immune responses. All piglets used for the ETEC F4 challenge were in good health and the growth of piglets treated with probiotics or antibiotics was comparable to that of the control piglets (unpublished results). These results were similar to those reported in a previous study [7].

Enteropathogens such as ETEC F4 must colonize the intestine to cause diarrhea. With regard to the development of ETEC F4 infection, attachment to the intestinal mucosa is the first step in the pathogenesis of diarrhea due to this pathogen [1]. The results of the present study showed that the administration of *P. acidilactici *or *S. cerevisiae boulardii *reduced ETEC F4 attachment to the ileal mucosa in comparison with the ATB treatment. The reduction of ETEC F4 adherence did not seem to be due to a probiotic bactericidal effect, given that the total ETEC F4 count in the ileum was not reduced. Moreover, little is known about the genes and external factors influencing F4 receptor expression. Nevertheless, it has been shown that diet can influence F18 receptor expression in pigs [17]. Therefore, we cannot exclude that probiotics could influence intestinal receptor expression such as F4, although this assumption is not yet supported in the literature. It seems more likely that *P. acidilactici *and *S. cerevisiae boulardii *reduced ETEC F4 attachment to the ileal mucosa through other mechanisms, such as competition, modulation of intestinal bacterial populations, and increased barrier function, that can impair the potential of ETEC F4 to bind to its F4 receptor. Indeed, an in vitro study demonstrated that *E. coli *attachment to porcine intestinal IPEC-1 epithelial cells is inhibited by probiotics [18]. Interestingly, when *P. acidilactici *and *S. cerevisiae boulardii *were administered together in our study, no significant difference in the attachment of ETEC F4 was observed, although there was a tendency to a decrease. This result suggests that there is no synergistic effect and possibly an antagonistic effect between the two probiotics with respect to their potential to reduce ETEC F4 attachment. In a previous study carried out with both probiotics [7] and in the present study [9], we also observed an antagonistic effect of *S. cerevisiae boulardii *on the capacity of *P. acidilactici *to modulate bacterial diversity after weaning. In both studies, ileal bacterial diversity was reduced in piglets treated with the *P. acidilactici *alone whereas this effect was abrogated when the two probiotics were administered together. Further studies are needed to elucidate the interaction between *S. cerevisiae boulardii *and *P. acidilactici *and its antagonistic effect on microbial populations in the gut.

Many studies carried out with different animal models, including pigs, also demonstrate the potential of probiotics to improve intestinal barrier function and reduce intestinal bacterial translocation [7,19]. In the present study, the probiotic treatments had no effect on ETEC F4 translocation through the intestinal mucosa, as determined by the ETEC F4 count in the MLN. This result contrasts with our previous observations indicating that the administration of antibiotics, *P. acidilactici*, *S. cerevisiae boulardii *and *P. acidilactici *+ *S. cerevisiae boulardii *to pigs reduces translocation of facultative anaerobic bacteria to the MLN, as compared to the control group, after a challenge with the ETEC F4 strain [7]. The difference in the results could be explained to some extent by the challenge model used in both studies, the age of the pigs, and the difference in the culture media used for the enumeration of bacteria. In the previous study [7], bacterial translocation was measured 24 h after three consecutive days of ETEC challenge in 52-day-old pigs, and the enumeration of bacteria in the MLN was performed using blood agar growth medium, which supports the growth of a wide range of organisms. In the present study, in contrast, a one-day challenge was used in 28-day-old pigs, and bacterial counts were performed 24 hpc using MacConkey agar No. 2 supplemented with nalidixic acid at 50 μg/mL, a medium that is selective for coliform organisms, specifically the Nal^r ^variant of the ETEC F4 strain used in this study.

In the literature, there is also clear evidence that certain probiotics reduce the severity of diarrhea caused by different pathogens, including *E. coli, Salmonella enterica *serovar Typhimurium, and rotavirus; these studies support the potential of probiotics to attenuate the host response to infectious organisms [20,21]. In the present study, although the accumulation of fluid in different sites of the intestine and the ETEC F4 count at the mucosal level were not affected by probiotic treatments, our results suggest that *P. acidilactici *and *S. cerevisiae boulardii *may interfere in certain conditions with ETEC F4 attachment to its receptor. In fact, the attachment of ETEC to specific F4 receptors in the ileum brings bacterial enterotoxins into close proximity with specific receptors on intestinal epithelial cells [1]. Moreover, other research recently showed in vitro that different strains of *Pediococcus *have the potential to down regulate Shiga toxin 2 gene expression in enterohemorrhagic *E. coli *O157:H7 [22]. In a further study, it would be interesting to determine whether *P. acidilactici *could modulate ETEC F4 enterotoxin gene expression and diarrhea severity over a longer period of time post-challenge.

Colonization of ETEC F4 and its attachment to the ileal mucosa are implicated in the stimulation of the host's innate immune response. In the present study, it was observed that ETEC F4 challenge stimulated cytokine expression and that probiotics could modulate the expression of cytokines involved in innate immune defence against ETEC F4. For instance, *P. acidilactici *+ *S. cerevisiae boulardii *significantly increased the expression of IL-6, a proinflammatory cytokine, in the ileum of the ETEC F4-challenged pigs. A tendency toward an increase in the expression of the IL-6 gene was also observed in the *P. acidilactici *group in comparison to the CTRL pigs but not in the *S. cerevisiae boulardii *group, suggesting that the effect obtained when the two probiotics were combined was primarily due to *P. acidilactici*. These results support those of another study reporting that probiotic bacteria such as *Bifidobacterium lactis *BB12 stimulated IL-6 production in primary murine intestinal epithelial cells [23]. In addition, this cytokine plays an important role in the regulation of intestinal immune response, barrier fortification, activation of neutrophils and B cell IgA isotype switching, important components in the defence against enteric infections [24]. Similarly, ileal TNF-α gene expression tended to be upregulated in the *P. acidilactici *+ *S. cerevisiae boulardii *group in comparison with the CTRL group, whereas there was no difference between the ATB and CTRL groups; these observations suggest that the administration of probiotics induced a stronger inflammatory reaction than the feeding of an ATB-enriched diet. However, these results also suggest that the presence of *P. acidilactici *was required to upregulate TNF-α gene expression as, in pigs treated with *S. cerevisiae boulardii *alone, expression of that gene was not increased and remained similar to that of the CTRL pigs. Other studies reported that lactic acid bacteria could induce innate cytokines, such as TNF-α, in leukocyte sensitized Caco-2 cells [25]. The expression of pBD-2, an antimicrobial peptide displaying broad antimicrobial activity against several pathogenic intestinal bacteria [26], and of IL-12p35, which is involved in the activation of mucosal innate immunity against enteric pathogens [27], also tended to be increased in the *P. acidilactici *group only in comparison to the CTRL pigs following ETEC F4 challenge. These results support in vitro studies indicating that lactobacillus strains and VSL#3, a probiotic cocktail of four lactobacilli, three bifidum and one streptococcus species, have the potential to induce the secretion of the BD-2 peptide by Caco-2 cells [3] and of IL-12 by human peripheral blood mononuclear cells [28]. As mentioned above, the trends observed in the present study in the expression of genes involved in innate immunity should not be ignored, because there are wide individual variations in the expression of these genes following exposure to infectious pathogens. Taken together, these results indicated that *P. acidilactici *either alone or in combination with *S. cerevisiae boulardii *had the potential to stimulate innate immune defence in ETEC F4-challenged pigs. However, further studies are required to evaluate whether the modulation of innate immune response by *P. acidilactici *is beneficial to the host in regard to controlling infections caused by ETEC F4.

A notable finding of this study was the significant increase in IL-8 gene expression in the ileum of the pigs challenged with ETEC F4 as compared to the non-challenged pigs. These results are in agreement with other in vitro studies that indicate an increase in IL-8 production following the stimulation of porcine intestinal epithelial cells, Caco-2 cells or a porcine macrophage cell line (3D4/31) with ETEC F4 [29-31]. Surprisingly, IFN-γ expression tended to be decreased in the ileum of the ETEC F4-challenged pigs in comparison to the non-challenged pigs. However, it was reported that oxidative or nitrosative stress occurring in different tissues is capable of inhibiting the production of IFN-γ, with that inhibition playing a key role in regulating early gene expression during gram-negative bacterial infection [32]. It is therefore possible that oxidative stress induced by ETEC F4 was still inhibiting the expression of IFN-γ at 24 hpc.

Finally, the different treatments in the non-challenged animals had no effect on the expression of ileal cytokines, with the exception of IL-8 and pBD-2. The increased IL-8 expression following the *S. cerevisiae boulardii*, *P. acidilactici *+ *S. cerevisiae boulardii *and ATB treatments is in contrast with in vitro results in human intestinal epithelial cells, where a different strain of *S. cerevisiae boulardii *exerted an anti-inflammatory effect by producing a low molecular weight soluble factor that blocks NF-κB-mediated IL-8 expression [33]. It could be hypothesized that *S. cerevisiae boulardii *interacts with molecules at the surface of intestinal epithelial cells, leading to IL-8 secretion in the present in vivo model, but further study is needed to confirm such a hypothesis. As an upregulation of this cytokine was not observed in the *P. acidilactici *animals, it can be assumed that the effect observed in the *P. acidilactici *+ *S. cerevisiae boulardii *pigs was primarily due to the action of *S. cerevisiae boulardii*. Moreover, there was a trend (although not statistically significant) toward an upregulation of pBD-2 expression in the non-challenged *S. cerevisiae boulardii *animals. The upregulation of antimicrobial peptides is a well-known mechanism of action for bacterial probiotics but a new one for yeast probiotics [3].

In conclusion, the administration of *P. acidilactici *or *S. cerevisiae boulardii *limited the attachment of ETEC F4 to the ileal mucosa, a key step in the pathogenesis of disease due to this pathogen. As expected, the antibiotics used in this study had no direct effect on ETEC F4 attachment or colonization since the *E. coli *strain showed acquired resistance to the antibiotics added to the weanling feeds in the ATB group (chlortetracycline and tiamulin). The antibiotics used in this study are commonly used by pig producers in North America in weanling feed to improve performance and to prevent enteric and respiratory diseases. Therefore, the ATB group was considered as a reference group. In addition, *P. acidilactici*, but not *S. cerevisiae boulardii*, influenced the expression of cytokines involved in the intestinal immune defence against ETEC F4. Taken together, these results indicated that *P. acidilactici*, through its potential to modulate intestinal immune functions and to influence host interaction with ETEC F4, could be a complementary approach to consider in strategies to improve intestinal health and reduce the use of antibiotics in weanling feed.

## Competing interests

The authors declare that they have no competing interests.

## Authors' contributions

JFD carried out microbial and immunological analysis, participated in the development of qPCR and drafted the manuscript. ML conceived of the study, participated in its design and coordination, supervised research activity throughout the project and participated in performing statistical analysis of data. FB supervised laboratory work and participated to microbial and immunological analysis. EN participated in the design of the study and was involved in the challenge protocol with *E. coli*. NB was involved in the development of qPCR assays for cytokines. YB participated to the development of qPCR assays and carried out immunological analysis. JPB and KL participated to sampling of tissues and microbial analysis. JMF participated in the conception of the study and in its design and coordination. All authors read and approved the final manuscript.
